# CMPO-Functionalized Silica Sorbents for pH-Tunable Separation and Enrichment of Rare-Earth Elements from Environmental Matrices

**DOI:** 10.1021/acssuschemeng.5c11109

**Published:** 2026-02-16

**Authors:** Ahmed K. Sakr, Sai Praneeth, Preetom K. Roy, Timothy M. Dittrich

**Affiliations:** Department of Civil and Environmental Engineering, 2954 Wayne State University, 5050 Anthony Wayne Drive, Detroit, Michigan 48202, United States

**Keywords:** solid−liquid extraction, critical minerals, chelating ligands, phosphate rock fertilizer, mesoporous silica, separation

## Abstract

Rare-earth elements (REEs) are crucial in many applications, yet mutual separation is challenging due to their similar chemical behavior. Octyl-phenyl-*N*,*N*-diisobutyl carbamoyl methyl phosphine oxide (CMPO) is an organophosphorus ligand originally developed for extracting actinides and lanthanides from spent nuclear fuel. Here, we report a pH-tunable CMPO-functionalized silica sorbent for selective REE separation from complex aqueous matrices. A CMPO-associated silica gel sorbent was synthesized and characterized by Brunauer–Emmett–Teller (BET) surface area, scanning electron microscopy, and X-ray photoelectron spectroscopy to confirm the surface functionalization and binding behavior. Sorbent performance was evaluated by using a synthetic 46-element solution and a real phosphate rock fertilizer leachate. Notably, REEs were successfully eluted with ultrapure water, demonstrating reversible desorption controlled by pH adjustment. Packed-bed column studies increased the REE mass fraction from 3.6% to 64% (20-fold enrichment), with up to 30-fold enrichment of neodymium. The adsorption process follows the Langmuir isotherm behavior and follows pseudo-second-order kinetics. The uptake capacity of 1 μmol of REEs per 4.2 μmol of CMPO supports the formation of a predominantly 4:1 ligand:rare earth element­(III) pseudocomplex. These results demonstrate CMPO-functionalized silica as a selective, water-elutable, and low-chemical-input platform for sustainable REE recovery from environmental and industrial sources.

## Graphical abstract

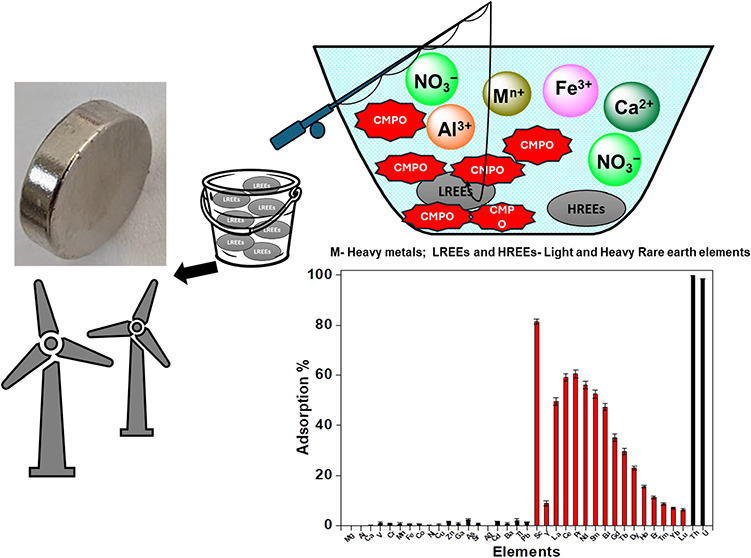

## Introduction

Rare-earth elements (REEs) are a group of 17 elements that include scandium, yttrium, and the lanthanide series. The predominant +3 oxidation state imparts distinctive magnetic, luminescent, and electrochemical properties.^1,2^ Despite their name, these elements are not particularly rare in the Earth’s crust. However, their extraction and processing are complex and often environmentally challenging.^3^ REEs are highly valued for their remarkable electrical and optical properties, making them essential components in cutting-edge technologies.^4^ REEs are seen as vital resources for the 21st century because of their crucial role in modern technology. A range of methods is employed to initially separate REEs into groups (e.g., heavy REEs, light REEs) and then further isolate individual elements to achieve high-purity rare earth oxides (REO).^5^

Solvent extraction, also known as liquid–liquid separation, is a common method for isolating and concentrating REEs from solution.^6^ It involves two immiscible liquids: (i) the aqueous phase is the acidic leachate containing dissolved REEs, and (ii) the organic phase is a nonpolar diluent with ligands dissolved to selectively coordinate with specific elements or REEs. The two phases are brought into contact and mixed to allow the REEs to interact with the ligands in the organic phase.^7^ The two liquid phases are then settled into two layers after the transfer of REEs (organic on top of the aqueous phase), and the process is repeated many times to obtain very pure REEs solutions. Although solvent extraction efficiently separates REEs,^8^ this technique includes expensive waste disposal due to large volumes of organic waste and operational difficulties such as the potential formation of a “third phase,” when the organic phase becomes oversaturated and forms a mixed organic-aqueous phase.

Solid–liquid separation processes present a potentially cost-effective and more eco-friendly alternative to liquid–liquid extraction.^9^ Solid–liquid separation involves simple steps, lower energy requirements, and efficient scalable production, along with less waste generation.^10^ Solid–liquid separation techniques have been extensively employed for adsorption of REEs from aqueous solutions using various organic, inorganic, and organic–inorganic adsorbents.^11,12^ Silica-based materials are widely employed for incorporating organic ligands because of their high versatility and ease of impregnation.^13^

Organophosphorus ligands play an important role in the recovery of REEs due to their distinctive chemical properties and versatility.^14–16^ Organophosphorus ligands can strongly coordinate with REEs in mixed solutions to form stable complexes and can precisely extract specific REEs from mixtures based on system designs. The chemical structure of organophosphorus ligands can also be readily modified to improve the extraction capacity, stability, specific REE selectivity, and environmental degradation potential.

Various organophosphorus ligands such as dinonyl phenyl phosphoric acid (DNPPA),^17^ 2-ethylhexylphosphonic acid mono (2-ethylhexyl) ester (PC-88A),^18,19^ di-2,4,4-trimethylpentyl phosphinic acid (Cyanex 272),^20,21^ di­(2-ethylhexyl)­phosphoric acid (D2EHPA),^22,23^ tributyl phosphate (TBP),^24–26^ and trioctyl phosphine oxide (TOPO)^27,28^ have been utilized in the separation and purification of REEs from high-level radioactive waste and mineral leachates. Organophosphorus compounds have also been incorporated in solid supports to form ion exchange resins used for the extraction of REEs in a solid–liquid separation system.^29,30^ However, there is a limitation of using these ligands in REEs separation because of their narrow acidity application range and poor selectivity.

Octyl-phenyl-*N*,*N*-diisobutyl carbamoyl methyl phosphine oxide (CMPO), also known as *N*,*N*-diisobutyl-2-[octyl­(phenyl)­phosphoryl]­acetamide, is a neutral organophosphorus ligand which has been developed to extract trivalent minor actinides in the TRansUranium Extraction (TRUEX) process.^31–36^ CMPO contains two active functional groups (P = O and C = O), enabling mono- or bifunctional extractant properties. Unlike other extractants, CMPO exhibits high efficiency during a wide acidity range (1–5 M HNO_3_), making it versatile for extraction process design and optimization.^37,38^ These noteworthy properties make CMPO a promising ligand for REE extraction media for aqueous solutions. The pioneering research of E.P. Horwitz also led to the development of commercially available chromatography resins that exploit CMPO coordination chemistry by impregnating porous styrene-divinylbenzene (PS-DVB) with CMPO and tri-*n*-butyl phosphate as a phase modifier (Eichrom, TRU resin).^39^ Although TRU resin is commonly used for environmental sample evaluation; limitations including high expense, small particle size, and difficulty in regeneration limit potential for more widespread use and industrial-scale rare earth element applications.

Yaftian et al. used diphenyl-*N*,*N*-dimethylcarbamoylmethylphosphine oxide, a CMPO derivative ligand, for solvent extraction of Eu^3+^ and Th^4+^ ions from nitric media.^40^ Their study reveals that the CMPO-type compound forms ML complexes of 1:2 and 1:3 for Eu^3+^ and Th^4+^ ions, respectively. Sengupta et al. investigated CMPO solvent extraction of Eu^3+^ ions from nitric medium and the effect of TBP or iso-decanol as modifiers.^41,42^ The study reported the stoichiometry of the metal–ligand complex as 3 molecules of CMPO associated with 1 cation of Eu^3+^. A spectroscopic study conducted by Gujar et al. investigated the complexation between Eu^3+^ ions and CMPO in ([C_4_mim]­[NTf_2_]) as a 1:3 complex,^43^ which is in agreement with the data reported previously by Sengupta et al.^41,42^ Conversely, Wu et al. studied the complexation between CMPO in the ionic liquid 1-butyl-3-methylimidazolium bis­(trifluoromethanesulfonyl)­imide ([C_4_mim]­[NTf_2_]) and Nd^3+^ ions and reported the formation of 1:4 [ML_4_]^3+^ complex.^44^

Research concerning the application of CMPO-functionalized solid supports for the separation and recovery of REEs has been relatively limited. Wei et al. reported a fixed-bed chromatographic separation of Y^3+^, Nd^3+^, and Gd^3+^ ions from a 3.0 M nitric solution containing Cs^+^, Sr^2+^, Ru^3+^, Y^3+^, Nd^3+^, and Gd^3+^ ions using a formylstyrene–divinylbenzene silica particles (SiO_2_/P) immobilizing CMPO extractant. The study confirmed the affinity of CMPO with REEs and that the sorbed REEs were effectively stripped by using water. A silica-based resin impregnated with CMPO-([C_8_mim]­[NTf_2_]) was used for the extraction of Am^3+^ and Eu^3+^ ions by Ansari et al.^45^ The uptake capacity for Eu^3+^ from a 3.0 M HNO_3_ was 18.95 ± 1.13 mg g^–1^ and determined that the sorption mechanism is chemisorption. Kumar et al., used a CMPO-functionalized microporous polymeric membrane for the separation of radio Eu^3+^ ions from a nitric media and showed that the separation of Eu^3+^ improved with increasing CMPO concentration.^46^ Gomez et al. incorporated a diethylphosphonoacetic acid, a CMPO derivative compound, in a mesoporous silica material for extraction of La^3+^ and Ce^3+^ ions from a neutral pH aqueous solution and reported the sorption capacity as 17.0 and 30.09 mg g^–1^ for La^3+^ and Ce^3+^ ions, respectively.^47^

To our knowledge, no published study has investigated the selectivity of CMPO for each REE in the presence of a mixed ion (>45 elements) aqueous solution. In our study, a novel CMPO-impregnated silica-based material was prepared and characterized for potential incorporation into an eco-friendly solid–liquid separation process. We report the selectivity of the CMPO-impregnated material toward the extraction of the 16 naturally abundant REEs from an actual leaching solution containing significant concentrations of major cations, heavy metals, and actinides. The effect of pH and solution chemistry on kinetics and sorption efficiency have also been studied. Finally, a packed-bed substitution and extraction chromatography technique was performed for individual separation of the REEs.

## Materials and Methods

### Chemicals and Instrumentation

The chemical ligand *N*,*N*-diisobutyl-2-[octyl­(phenyl)­phosphoryl]­acetamide-“CMPO” (CAS#: 83242–95–9) was purchased and used as received (95% purity; AmBeed, Arlington Heights, IL). The CMPO was stored in the freezer upon receipt and brought to room temperature prior to use in media preparation. Two solid supports were utilized in the study: chemically modified organosilica (60–80 mesh) obtained from ABS Materials (Wooster, OH) and high-purity silica gel (70–230 mesh) from Supelco, purchased through Sigma-Aldrich.

A 17-component standard solution containing 16 rare earth elements (REEs) and thorium (Th) in 2% HNO_3_ was sourced from High Purity Standards (Charleston, SC). The concentration of each element in the solution is 100 mg L^–1^; this solution was used to prepare synthetic REEs and Th solutions equivalent for experimental use by measuring CMPO capacity and selectivity. High-purity nitric acid (Fisher Chemical, OPTIMA grade) was employed for adsorption studies and inductively coupled plasma mass spectrometry (ICP-MS) sample preparation. Methanol (Fisher Chemical, Optima) was used to dissolve CMPO before impregnation, while aliphatic kerosene (Fisher Chemical) was used as a diluent in solvent extraction experiments. Aliphatic kerosene was chosen as the diluent for CMPO due to its chemical inertness, low aqueous solubility, and ability to reduce viscosity improving mass transfer and complexation with REEs. Kerosene is also cost-effective, widely available, and commonly used in industrial-scale solvent extraction. All chemicals were ACS-grade and used without further purification.

Sample measurements were conducted by using precise laboratory instruments. Analytical weighing was performed with a Mettler-Toledo MS304TS balance (Greifensee, Switzerland) with a readability of 0.1 mg. Aqueous REEs concentrations were analyzed using Agilent 7850 ICP-MS (Santa Clara, CA). Sample pH was determined using a Fisher Scientific Accumet AE150 benchtop pH meter equipped with a pH-specific electrode. Mixing of samples was achieved using a Fisher Scientific multipurpose tube rotator, operating at speeds ranging from 5 to 80 rpm.

Sorbent materials were characterized in the Lumigen Instrument Center at Wayne State University. Scanning electron microscopy (SEM) was performed using a JEOL JSM-7600F instrument (Tokyo, Japan) with integrated energy dispersive X-ray spectroscopy (SEM-EDS; Pegasus Apex 2) to analyze surface morphology. X-ray photoelectron spectroscopy (XPS) analyses were performed using a ThermoFisher Scientific NEXSA (Waltham, MA), equipped with a monochromated Al K-α 1486 eV X-ray source and Avantage software to determine elemental composition. Sorbent surface area, pore size, and pore volume were determined using the Brunauer–Emmett–Teller (BET) N_2_ gas adsorption–desorption technique (Micromeritics TriStar II 3020, Micromeritics Instrument Corporation, Norcross, GA).

### Preparation of CMPO-Impregnated Silica and Organosilica Sorbent Media

Organosilica and silica solid supports were loaded with 20% (w/w) CMPO following modified methods described by Praneeth et al.^48^ Briefly, CMPO is dissolved in methanol and rotated with the solid support for 1–2 h. Organic solvents were then removed from the CMPO-impregnated media using a vacuum centrifuge concentrator (Vacufuge Plus concentrator; Eppendorf; Hamburg, Germany) with a methanol recovery reservoir. The dried media was stored in the air-sealed container and utilized for subsequent experiments. Two different sorbents (CMPO-functionalized silica gel and CMPO-functionalized organosilica) were initially synthesized to evaluate their potential use in advanced solid–liquid extraction systems. Preliminary performance screening demonstrated several advantages of the CMPO-functionalized silica over organosilica (including >3.8 times higher binding capacity the silica solid support), and was selected as the focus of the advanced characterization and proof-of-concept experiments.

CMPO ligand baseline complexation values in hydrochloric, nitric, and sulfuric acid solutions were measured via solvent extraction (SX). The effect of acidic medium (HCl, HNO_3_, and H_2_SO_4_) on REEs extraction and selectivity was investigated by using a solvent extraction (SX) separation method to determine baseline complexation values. The extraction was carried out in 50 mL polypropylene tubes using equal volumes of (20 mL) the aqueous (varying HCl, HNO_3_, and H_2_SO_4_ containing 102 mg L^–1^ of the 16 REEs + Th) (6.0 mg L^–1^ for each of the 17 elements) and organic phase (kerosene with 20 mM of CMPO dosage) in 1.0 M of respective acids. The aqueous/organic mixture was transferred to a 60 mL separation funnel after 15 min of contact time at 50 rpm. Separated phases were filtered, diluted, and analyzed by ICP-MS for metal concentration. Equations are detailed in Text S1.

### Batch Sorption Experiments (Capacity, Kinetics, Solid Support Type, 1–5 M Acid Range, 43-Element Competition, Phosphate Rock Leachate)

Batch studies were conducted by adding CMPO sorbent media (150 mg) to centrifuge tubes (50 mL, polypropylene) with a 17-element synthetic solution (36 mL) for a test dosage of 3.0 g L^–1^. Tubes were rotated (10 rpm, 24 h) at ambient temperature (25 °C ± 1) unless otherwise stated. The suspension was filtered (0.2 μm syringe filter, Basix, Fisher Scientific, nylon), diluted into 2% nitric acid, and measured for REE concentration by ICP-MS. A synthetic solution containing 17 elements was prepared for this study. Individual concentrations of 6.0 mg L^–1^ for 17 elements (16 rare-earth elements and thorium) were achieved by diluting an initial stock solution (HPS, ICP-MS standard).

The influence of solid support on the potential extraction and selectivity of REEs through batch sorption experiments was evaluated with 10% and 20% CMPO loading (wt %). Various factors were investigated to optimize the sorption process. Each experiment was conducted in duplicate, and the average and standard deviation were calculated and presented. Sorption experiments were performed over a range of nitric acid concentrations (1.0–5.0 M HNO_3_) with 3.0 g L^–1^ dosage with 6.0 mg L^–1^ of individual REE concentration to determine the role of acid strength. Kinetic studies were conducted by measuring sorption rates at contact times from 2.0 min to 24 h in 1.0 M HNO_3_ and the data were fitted to pseudo-first order and pseudo-second-order kinetic models with equations presented in Text S2.

The sorption competition in the presence of other major ions was carried out by preparing a solution containing Ag, Al, As, B, Ba, Be, Ca, Cd, Ce, Co, Cr, Cs, Cu, Dy, Er, Eu, Fe, Ga, Gd, Ho, K, La, Lu, Mg, Mn, Na, Nd, Ni, P, Pb, Pr, Rb, S, Se, Sm, Sr, Th, Tl, Tm, U, V, Yb, Zn, Sc, Y, and Tb. This solution was prepared by multielement standard containing 43 elements (IV-ICPMS-71A, Inorganic Ventures, 10 mg L^–1^ each of 43 elements) in nitric acid (3% v/v) and Sc, Y, Tb (calculated for standard concentration) addition to the solution.

The sorption efficiency (*E*, %) and sorption capacity *q*_e_ (mg g^–1^) were calculated using eqs 1 and 2:

1 $$E\left(\%\right)=\frac{C_{0}-C_{e}}{C_{0}}\times 100$$

2 $$q_{e}=\left(C_{0}-C_{e}\right)\times \left(\frac{V}{m}\right)$$

where *C*_0_ and *C*_e_ (mg L^–1^) are the initial and equilibrium REEs concentrations, respectively, *V* (L) is the solution volume, and *m* (g) is the mass of sorbent material.

Additional details of experiments testing acid range, solid support type, kinetics, capacity, and competition of co-ions are presented in Text S2 and desorption studies in Text S3.

### Packed-Bed Column Experiment

A column experiment was conducted to demonstrate the feasibility of individually separating REEs from a mixed stock solution with the new CMPO media, as outlined in previous studies.^49,50^ CMPO-silica gel media (113 mg) was dry-packed into a small borosilicate column (Cole-Parmer, BENCHMARK, 3 mm inner diameter, 2.5 mm length).^48^ Before the experiment, the column was flushed with 1.0 M nitric acid to prepare the media. A mixed solution containing 102 mg L^–1^ of REEs, 6.0 mg L^–1^ each REEs + Th, under 1.0 M nitric acid medium, was introduced into the column using a peristaltic pump from the bottom to reduce the risk of air bubble formation. The experiment was conducted at a constant flow rate of 0.3 mL h^–1^ with a void volume of 0.096 mL. Effluent from the column was collected by using a fraction collector. Test tubes were weighed and sampled for ICP-MS analysis within 8 h of collection. Once a breakthrough occurred (indicated by *C*/*C*_0_ > 0.5), the column media was stripped with high-purity water and the collected fractions were subsequently analyzed using ICP-MS.

### Proof-of-Concept REE Recovery from a Phosphate Rock Leachate

Batch studies were also conducted with a phosphate rock leachate (Table S3), with leaching conditions (1 M nitric acid, 24 h, 1:20 g per ml solid to liquid ratio) similar to Tummala et al.^51^ and experimental conditions matching the 43-element solution described above.

## Results and Discussion

### Effect of the Solid Support Type

CMPO-impregnated silica gel and CMPO-impregnated organosilica were prepared and utilized for solid–liquid separation of the 16 REEs + Th from 1.0 M nitric acid solution in a screening experiment to determine the best solid support for advanced characterization and proof-of-concept separation experiments. As illustrated in Figure 1a, the effect of silica gel physically bonded with different amounts of CMPO was investigated. The sorption efficiency of total REEs onto the 10% CMPO-impregnated silica gel is 15.4%, and the sorption capacity of the media is 4.75 mg g^–1^. The sorption efficiency rose to 32.9% when 20% CMPO-impregnated silica gel was used for the adsorption process, and the sorption capacity became 8.94 mg g^–1^. The new CMPO-impregnated silica gel material shows a remarkable selectivity for light REEs (Sc, La–Eu) compared to heavy REEs (Y, Gd–Lu). Sc is the most noticeably sorbed element, whereas Lu exhibits the lowest sorption.^52^

**Figure 1:**
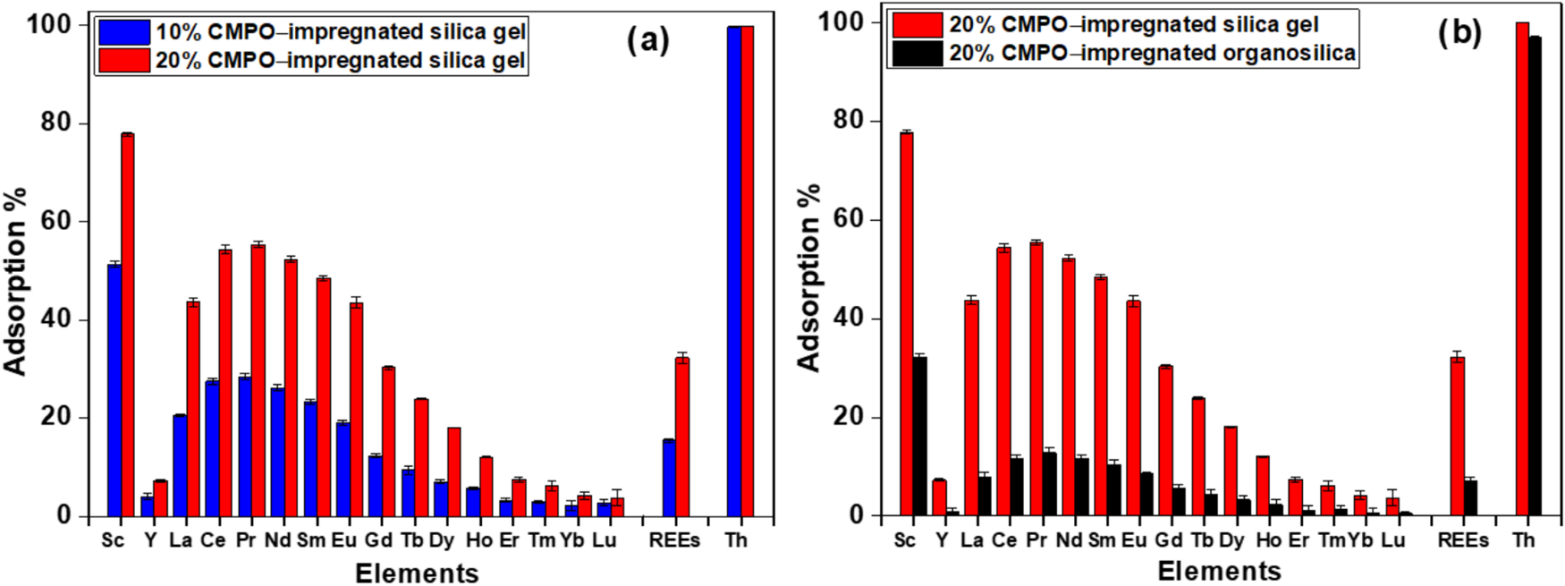
(a) Effect of concentration of CMPO incorporated on silica gel on the sorption of REEs (1.0 M HNO_3_, 102 mg L^–1^ REEs + Th, 24 h, 3 g L^–1^, 10 rpm, 25 °C) and (b) effect of solid support acid on the sorption of REEs (1.0 M HNO_3_, 102 mg L^–1^ REEs + Th, 24 h, 3 g L^–1^, 10 rpm, 25 °C).

Figure S1 shows the sorption experiment of the 16 REEs + Th on the silica gel, indicating that REEs were only sorbed because of CMPO immobilized in the silica gel. The preference of CMPO toward REEs + Th at equilibrium sorption based on equal mass concentrations is Th > Sc > Pr > Ce > Nd > Sm > La > Eu > Gd > Tb > Dy > Ho > Er > Y > Tm > Yb > Lu. The higher affinity of CMPO-impregnated silica gel for the lighter REEs group compared to the heavier group differs from diglycolamide affinity, especially TODGA, which has higher selectivity for heavy REEs, as reported by Praneeth et al.^53^ The selectivity of CMPO toward light REEs (Sc, La–Eu) over heavy REEs (Y, Gd–Lu) is more likely due to larger coordination numbers (9, 10) of light REEs, while heavy REEs trend to lower coordination number of 8.^52^ Furthermore, the ionic radii of (Sc, La, and Eu) are preferable in binding geometry with CMPO.

More than 99% of Th was sorbed on the 10% and 20% CMPO-impregnated silica gel. This is likely attributed to the higher charge +4 and the tendency of Th toward higher coordination numbers of 10 compared to the trivalent REEs^3+^,^54,55^ which increases the electrostatic attraction to O atoms of CMPO, facilitates the coordination with the bifunctional groups, phosphoryl (P = O) and carbonyl (C = O), of the CMPO, and forms a strong bidentate coordination between Th^4+^ ions and CMPO ligand as a 1:3 complex of Th­(NO_3_)_4_.3­(CMPO).^38,40,56^

Figure 1b compares REE sorption for 20% CMPO-functionalized silica gel and 20% CMPO-functionalized organosilica with 32.87% REE sorption onto the CMPO-functionalized silica gel (8.94 mg g^–1^ sorption capacity) and only 7.15% sorption onto the CMPO-functionalized organosilica (2.31 mg g^–1^ sorption capacity). As reported in previous work by Hovey and Dardona et al.,^57^ the unfunctionalized organosilica does not have any measurable affinity for REEs. Notably, however, the two sorbents exhibit the same behavior of affinity toward light REEs (Sc, La–Eu) over the heavy REEs (Y, Gd–Lu). The silica gel has a smaller particle size of 63–200 μm compared to the large particle size of 177–250 μm for the organosilica, leading to a larger surface area for the silica gel likely allowing more homogeneous and efficient coating of CMPO extractant on the surface.

REEs adsorption screening experiment results show a >3.8 times higher total REEs binding capacity for the silica solid support media compared to organosilica solid support. 32.9% of total REEs sorbed onto the CMPO-functionalized silica gel (8.9 mg g^–1^ sorption capacity), with only 7.2% sorption onto the CMPO-functionalized organosilica (2.3 mg g^–1^ sorption capacity). The sorption data presented in Figure 1b demonstrate successful initial impregnation of CMPO on two different silica-based solid support and consistent CMPO binding behavior (order and capacity) toward REEs; however, CMPO-silica was selected as the focus of the advanced characterization (SEM-EDS, surface area analysis, and XPS) and proof-of-concept experiments based on the superior binding capacity.

### SEM-EDS Characterization

Ligand attachment for functionalized sorbents usually falls into three categories; (i) covalent grafting (often via silane linkage), (ii) physical impregnation (physisorption), or (iii) incorporation in a co-condensate. Sorbents presented here were made via physical impregnation, with physisorption forces leading to adhesion/attachment, including H-bonding, π–π bonds, and van der Waals forces.

The surface morphology and elemental composition of silica gel particles and CMPO-impregnated silica gel media (before and after CMPO attachment) were analyzed using SEM-EDS, as shown in Figures S2 and S3. The SEM image of silica gel (Figure S2) reveals a uniform distribution of particles with sizes ranging from 50 to 200 μm. The EDS analysis presented in Figure S2b,c indicates that the primary components of the silica gel are silicon and oxygen, with an average weight percentage (wt %) of 54.6 and 45.4%, respectively.

SEM-EDS analysis of the CMPO-impregnated silica gel media is shown in Figure S2. The SEM image (Figure S3a) demonstrates that the surface morphology and particle size remained consistent with the original silica gel, confirming the structural stability of the particles after CMPO attachment. The EDS spectra from three random sample points (Figure S3b,d) reveal the presence of nitrogen (N) and phosphorus (P) in addition to silicon (Si) and oxygen (O), corresponding to the phosphoryl (P = O) and amide (N–C = O) groups from the CMPO molecules. The average wt % obtained from these points shows Si, O, N, and P at 61.8, 29.9, 1.5, and 2.6%, respectively, further validating the successful attachment of CMPO onto the silica gel.

### Surface Area Analysis

The surface areas of silica gel and CMPO-impregnated silica gel were determined with gas adsorption–desorption isotherms according to the Brunauer–Emmett–Teller theory (BET) method, and the Barrett–Joyner–Halenda (BJH) method was used to calculate the pore size and pore volume measurements. The data listed in Table 1 demonstrate an evident decrease in the values of surface area, pore volume, and pore size after immobilization of CMPO in silica gel. The specific surface area of CMPO-impregnated silica gel is reduced by more than 46% (from 450 to 240 m^2^ g^–1^) and the pore volume was decreased by almost 50% (from 0.67 to 0.34 cm^3^ g^–1^), while the reduction in the average pore size is <10% (from 55.9 to 50.5 Å). The decrease in these parameters indicates the successful immobilization of CMPO molecules in the pores and on the surface of the silica gel.

**Table 1:** Surface Area, Pore Volume, and Pore Size of Silica Gel and CMPO-Impregnated Silica Gel

sample	surface area (m^2^ g^–1^)	pore volume (cm^3^ g^–1^)	pore size (Å)
silica gel	450.01 ± 1.04	0.70	55.9
CMPO-impregnated silica gel	239.80 ± 1.35	0.34	50.8

### XPS Characterization

The XPS analysis was performed to identify the elemental composition of the media. The XPS spectrum of silica gel is given in Figure 2a; it shows two main peaks at 103.68 and 533.14 eV due to the binding energy of Si_2_ *p* and O_*1S*_, respectively. It is noteworthy to indicate that the unlabeled features between 978–1013 eV are due to the O_KLL_ Auger peaks. Figure 2b demonstrates the XPS spectrum of the CMPO-impregnated silica gel. Three new peaks are observed; one medium peak at 284.78 eV is attributed to C_1S_, accompanied by two weak peaks for N_1S_ and P_2p_ at 399.33 and 132.32 eV,^58^ respectively. The high resolution spectra of the C_1S_, N_1S_, and P_2p_ peaks are presented in Figure 2c–e. These peaks are due to the phosphoryl (P = O) and amide (N–C = O) groups of the CMPO molecules. This result indicates the successful incorporation of CMPO into the silica gel forming a new sorbent.

**Figure 2:**
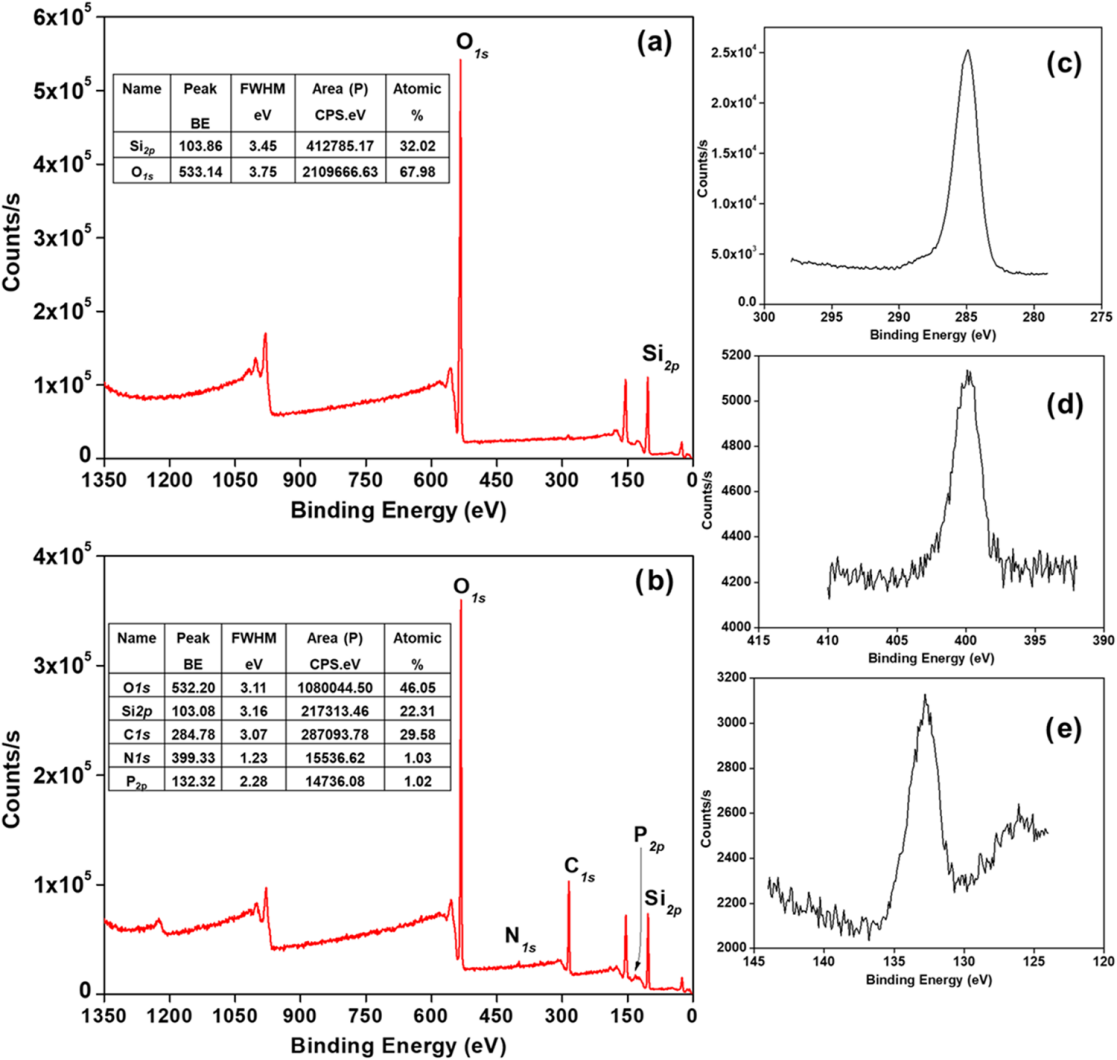
XPS spectra of (a) silica gel, (b) CMPO-impregnated silica gel, (c) C_1S_ in CMPO-impregnated silica gel, (d) N_1S_ in CMPO-impregnated silica gel, and (e) P_2p_ in CMPO-impregnated silica gel.

XPS was used to survey the sorbents for P and N peaks, and the Si/O/C envelope is a useful initial evidence that CMPO (which contains P and O) is present. These survey scans, however, do not prove bonding mode, stoichiometry, or depth distribution. Additional high resolution XPS (P_2p_, N_1s_, C_1s_, Si_2p_) with peak fitting, binding-energy comparisons, and quantitative analysis (atomic% or P/Si ratio) would be useful for more complete characterization. XPS can not be used alone to provide definitive information about CMPO incorporation. Cycling experiments also show strong indirect evidence of successful incorporation, as they integrate many complex variables into simple and direct proof of initial and reusable attachment.

### Sorption Study

Various parameters have been studied and optimized, including the type of acids, type of solid support, acid concentration, kinetics, isotherm models, and sorption competition of ions in the solution.

### Type of Acid

The effect of different acidic media on the behavior of CMPO during the extraction process of REEs has been investigated. As depicted in Figure S4, 25.08% of the total REEs in nitric acid medium has been extracted using CMPO, while only 2.35% of the total REEs was extracted from the hydrochloric acid media and only 1.26% of Sc was extracted from the sulfuric acid solution. Moreover, the affinity of CMPO in nitric acid solution is considerably higher for light REEs (Sc, La–Eu) with 58.10% than 41.89% for heavy REEs (Y, Gd–Lu) and a separation factor of 2.17, suggesting a notable selectivity for the lighter REEs group.

Nitric acid clearly affects the CMPO sorption behavior when compared to both hydrochloric acid and sulfuric acid. CMPO forms a stable 1:1 complex of CMPO.HNO_3_ with nitric acid, where the oxygen atom of the phosphoryl group in CMPO acts as a Lewis base and is protonated by nitric acid.^59^ As a result of this interaction, the electron density in the π–bond of the P = O bond in the CMPO is diminished, the polarization of the σ–bond increases, and consequently, the phosphorus atom becomes more deshielded. Hydrochloric acid and sulfuric acid are not able to form stable complexes with CMPO.

The presence of nitric acid in the aqueous phase plays a crucial role in resisting dealkylation of CMPO during the solvent extraction process, promoting the formation of stable metal complexes. The degradation of CMPO at the C–N bond of the amide group proceeds more rapidly in aqueous solutions containing chloride Cl^–^ or sulfate SO_4_^2–^ ions compared to the presence of nitrate NO_3_^–^ ions.^60^ The formed byproducts negatively impact the metal extraction process.

### Effect of Acid Concentration

The influence of nitric acid concentration on REE extraction with CMPO-impregnated silica gel was studied over concentrations ranging from 1.0 to 5.0 M HNO_3_, as present in Figure 3. The change in sorption efficiency of the total REEs is negligible, while Th was fully absorbed. The uptake capacity of the CMPO-impregnated silica gel is about 8.94 mg g^–1^. The sorbed amounts of La, Ce, Pr, Nd, Sm, and Eu were reduced from 43.69, 54.36, 55.71, 52.29, 48.47, and 43.58% at the 1.0 M HNO_3_ solution to 23.32, 32.89, 34.66, 33.87, 35.98, and 35.38% at the 5.0 M HNO_3_ solution, respectively. Nonetheless, the sorption efficiency was elevated from 7.25 to 19.63% for Y, 23.94 to 28.78% for Tb, 18.02 to 28.12% for Dy, 12.04 to 25.09% for Ho, 7.43 to 23.57% for Er, 6.12 to 22.33% for Tm, 4.18 to 20.17% for Yb, and 3.67 to 15.37% for Lu, when the HNO_3_ concentration in the solution augmented from 1.0 to 5.0 M, respectively. The main conclusion is that increasing the nitric acid concentration does not affect the CMPO-impregnated silica gel sorption capacity. Instead, the CMPO-impregnated silica gel sorbent became less selective at higher nitric concentrations, and the highest selectivity for light REEs was attained at 1.0 M HNO_3_ solution, which was used for further experiments.

**Figure 3:**
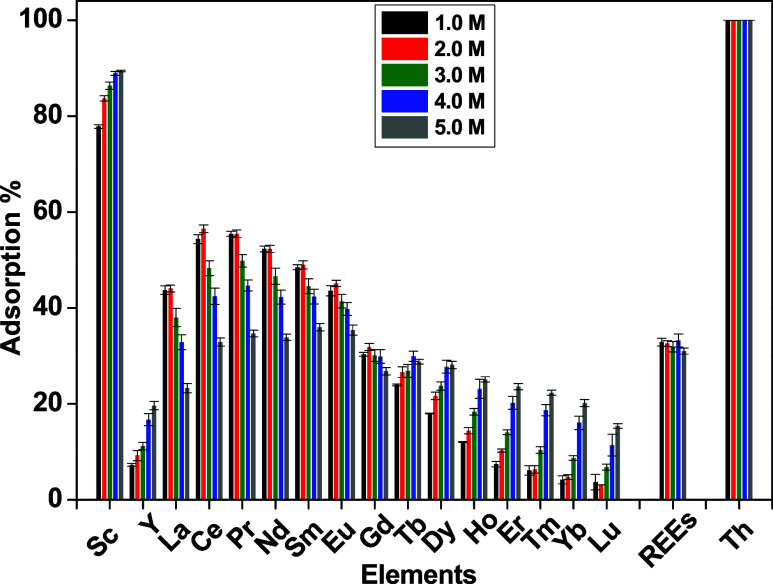
Effect of HNO_3_ on the sorption of REEs on the CMPO-impregnated silica gel (102 mg L^–1^ REEs + Th, 24 h, 3 g L^–1^, 10 rpm, 25 °C).

### Kinetics

The influence of time on REEs sorption to CMPO-impregnated silica gel was investigated in a 1.0 M HNO_3_ solution from 2.0 min to 24 h. As shown in Figure 4a, two steps are observed in the kinetics experiment with very rapid sorption of ∼90% of the capacity being reached in the first 5 min with additional sorption occurring until 4 h. After that, equilibrium is achieved, and sorption efficiency remains stable (∼32.87% of the total). Further studies used this 4.0 h equilibrium sorption time.

**Figure 4:**
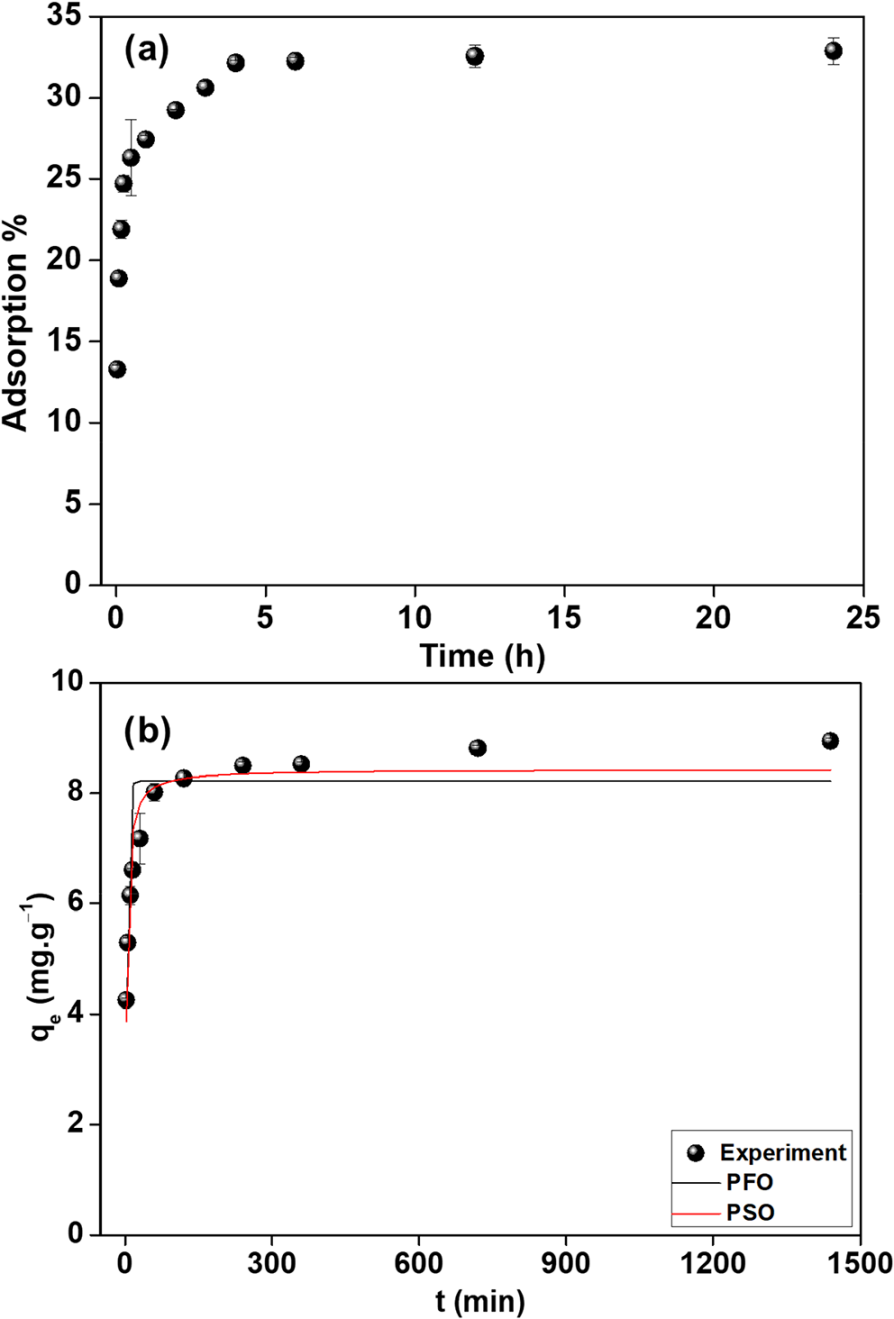
(a) Effect of sorption time (kinetics) for REEs and CMPO-impregnated silica gel (1.0 M HNO_3_, 102 mg L^–1^ REEs + Th, 3 g L^–1^, 10 rpm, 25 °C) and (b) kinetic models of REEs sorbed on CMPO-impregnated silica gel.

The pseudo-first-order (PFO) and pseudo-second-order (PSO) models were applied to describe the kinetic mechanism of the adsorption process. Figure 4b illustrates the fitting of PFO and PSO models with the kinetic data, while the kinetic parameters are tabulated in Table S1. The reduced chi-square (Χ2) value of the PSO model (96.6) is lower than that of the PFO (387.9). In addition, the PSO model exhibits a high correlation coefficient (*R* ^2^ = 0.95), which is close to unity and implies that the PSO kinetic model provides a more precise representation of the sorption of REEs on CMPO-impregnated silica gel sorbent. In addition, the presence of CMPO:REE pseudocomplex formation could also be responsible for a minor second binding (or association) type that is not reflected in the PSO model.

### Isotherms

The study investigated how varying the initial concentration of REEs in the 1.0 M HNO_3_ solution influences the sorption capacity and affinity of CMPO-impregnated silica gel. Different initial REE concentrations varying from 1.0 to 20 mg L^–1^ for each element were tested. As shown in Figure 5a, the sorption capacity of the CMPO-impregnated silica gel increased gradually from 3.03 to 10.74 mg g^–1^ with higher initial concentrations (1.0 to 9.0 mg L^–1^ of each element or 16 to 144 mg L^–1^ of total REEs). The higher concentration of REEs generates a robust mass transfer driving force, which raises the REEs sorption capacity of the CMPO-impregnated silica gel. Further increase of the initial concentration of each element to 20 mg L^–1^ (320 mg L^–1^ of total REEs) shows a marginal increase in the uptake capacity of the media. The maximum uptake capacity is 11.43 mg g^–1^. The CMPO-impregnated silica gel maintained a higher affinity for light REEs over heavy REEs, independent of the increases in the initial concentration (Figure 5b).

**Figure 5:**
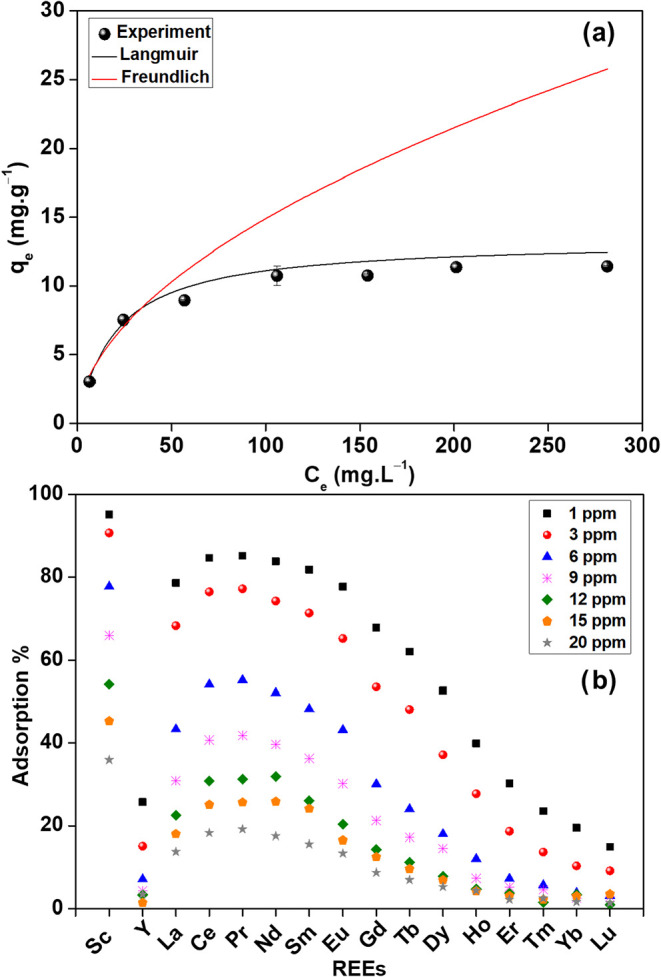
(a) Isotherm models of REEs sorbed on the CMPO-impregnated silica gel and (b) adsorption % of REEs on the CMPO-impregnated silica gel at different initial concentrations (1.0 M HNO_3_, 4.0 h, 3 g L^–1^, 10 rpm, 25 °C).

To identify the sorption mechanism of REEs on the CMPO-impregnated silica gel, Langmuir and Freundlich isotherm models were tested. The calculated isotherm data are listed in Table S2. The Langmuir isotherm model fits the experimental data well (*R* ^2^ = 0.99) compared to the Freundlich model (*R* ^2^ = 0.88), as shown in Figure 4a. Moreover, the value of *q*_m_ (13.35 mg g^–1^) is within 15% of the experimental value (11.43 mg g^–1^). The Langmuir isotherm model assumes a monolayer chemisorption mechanism of REEs on CMPO-impregnated silica gel, which is consistent with ligand complexation. The Langmuir isotherm is characterized using the dimensionless constant (*R*_L_) which was calculated by the following equation:

3 $$R_{L}=\frac{1}{{1+(K_{L}C}_{0})}$$

where *C*_0_ (mg L^–1^) is the highest initial concentration of total REEs used. The calculated *R*_L_ value was 0.059 and favorable sorption processes occur when 0 < *R*_L_ < 1.0.

The REEs uptake capacity was calculated as 0.0042 mmol of REEs per 0.0178 mmol of CMPO in the sorbent for a calculated CMPO/REE ratio of 4.2:1. The stochiometric analysis suggests the coordination of 1 REE^3+^ ion with 4 molecules of CMPO forming a 1:4 metal–ligand complex of REE­(NO_3_)_3_.4­(CMPO), which is consistent with the literature data reported previously by Wu et al.^44^ and Troxler et al.^61^ Figure 6 illustrates the coordination of one REE^+3^ ion with four CMPO molecules by forming bonds with two functional groups per CMPO. As reported by Troxler et al.,^61^ the metal-ligand complex likely consists of phosphoryl (P = O) and carbonyl (C = O) moieties from three CMPO molecules, along with an additional phosphoryl group (P = O) from a fourth CMPO molecule. These data contradict with the hypothesis of the 1:3 metal–ligand complex of Ce­(NO_3_)_3_.3­(CMPO) reported by Nakamura and Miyake^62^ and Eu­(NO_3_)_3_.3­(CMPO) reported by Sengupta et al.^42^

**Figure 6:**
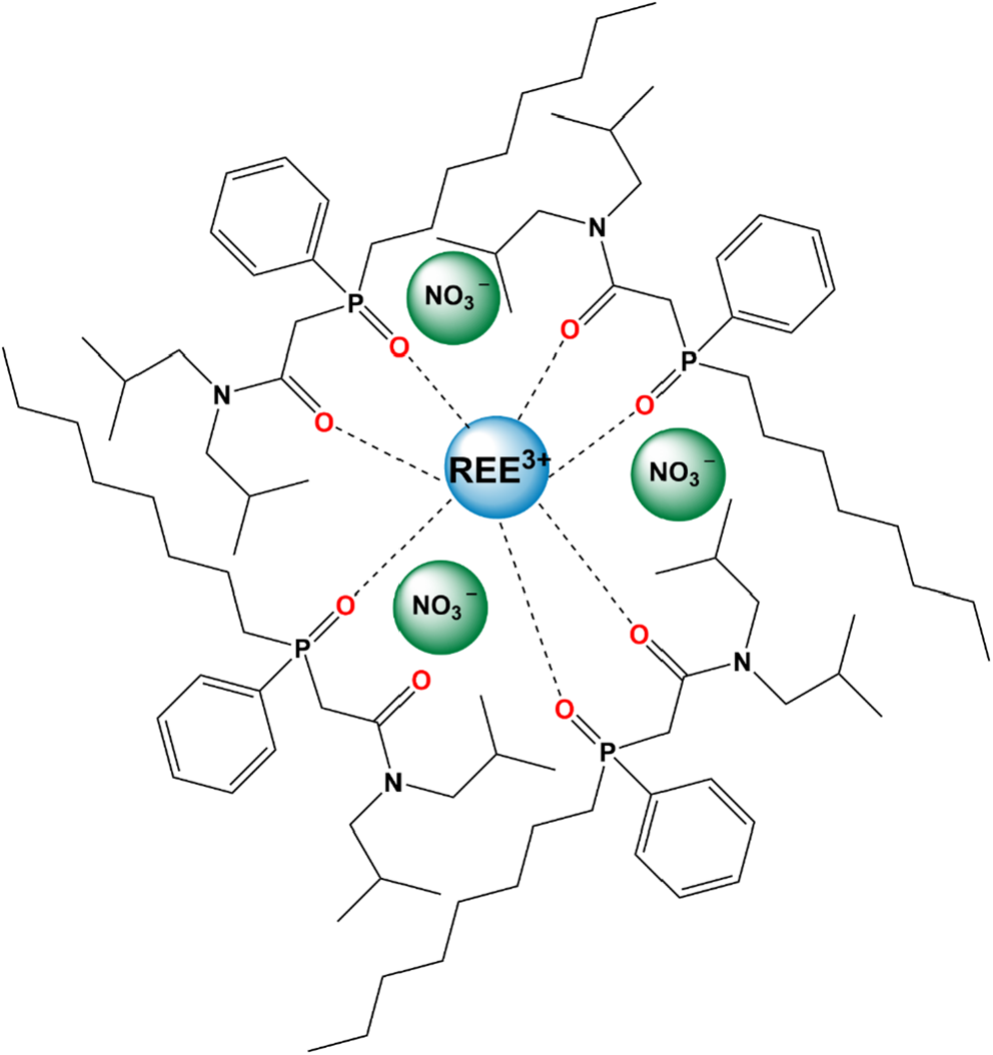
Illustration of the potential 1:4.2 REE-CMPO pseudocomplex in CMPO-impregnated silica gel. The proposed concept illustrates a pseudocomplex that includes one REE ion, three nitrate ions, and four CMPO molecules (three CMPO for two bonds, while one CMPO only bonds with the phosphoryl group and not the carbonyl), as has been proposed.^41,58^

Many factors affect comparisons between ligand binding in the solvent and solid phase extraction systems. In confined spaces within solid supports, the ligand concentration, solvent polarity, and ion pairing differ from those of bulk organic phases. The observed CMPO/REE ratio of ∼4.2:1 derived from sorption measurements does not necessarily imply the formation of a discrete inner-sphere 4:1 complex. The apparent CMPO/REE stoichiometry exceeding classical coordination limits is chemically reasonable when interpreted in terms of an outer-sphere pseudocomplex, in which one or two CMPO molecules coordinate directly to the REE ion, while additional CMPO molecules associate through outer-sphere electrostatic interactions, hydrogen bonding to coordinated nitrate or water, and ligand–ligand aggregation. Such pseudocomplexes are well documented in solvent extraction systems involving CMPO and TODGA and were reported by the Horwitz team in work with CMPO and the TRUEX system. The noninteger association number is an important distinction in comparing stoichiometry between pseudocomplexes and true (or structural) complexes. Spectroscopic techniques such as EXAFS primarily probe short-range, well-defined coordination environments and are therefore insensitive to outer-sphere or second-shell ligand association.

Although other organophosphate ligands such as TODGA form M­(NO_3_)_3_.3L complexes in both solvent extraction and solid–liquid separation, the coordination between REEs and CMPO for solvent and solid–liquid separations shows discrepancies. For rare earth elements, both CMPO and TODGA form stable 1:1 and 1:2 inner-sphere complexes. Evidence for higher stoichiometries (≥1:3) is largely indirect and better explained by outer-sphere association and/or aggregation, rather than true coordination complexes. Further studies are needed to better understand the stoichiometry and complexation mode of REEs with CMPO.

### Competition with Co-Ions

Although CMPO shows significant promise in the 16 REEs systems, the affinity of CMPO-impregnated silica gel for light REEs in the presence of non-REE metal ions (as are often found in leachate solutions) must also be evaluated. Figure 7 shows a tremendous selectivity for light REEs (Sc, La, and Eu) as well as Th and U, with slight uptake of heavy REEs (Y, Gd, and Lu) and distinctly negligible uptake of major ions. The result emphasizes the affinity of CMPO-impregnated silica toward light REEs (Sc, La–Eu) and the ability to extract light REEs directly from nitrate-derived leach liquors.

**Figure 7:**
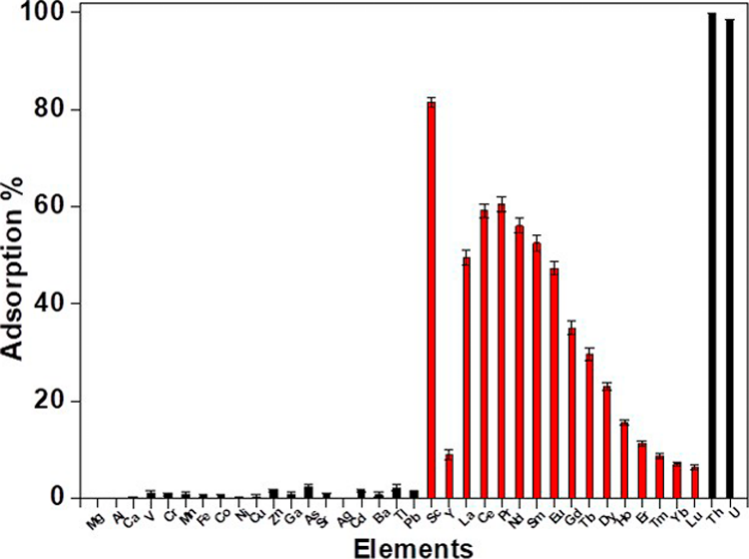
Effect of co-ions in solution on the sorption of REEs on the CMPO-impregnated silica gel (1.0 M HNO_3_, 4.0 h, 3 g L^–1^, 10 rpm, 25 °C). REEs are shown in red and other metals are shown in black.

### Desorption Study

REE desorption from the loaded CMPO-impregnated silica gel sorbent was investigated to determine the optimal release conditions, REE recovery yields, and the potential to reuse the media for repeat cycles.

### Type of Stripping Agent (Eluents)

Ultrapure water, NaHCO_3_, NaOH, and NH_4_OH were tested as stripping agents (or eluents). After 30 min of contact time in 1:40 S/L solutions, Figure S5a shows that ultrapure water attained the highest REE elution efficiency (42.4%), followed by NaHCO_3_ (31.0%), while NH_4_OH and NaOH released <5% of sorbed REEs. Water is a highly polar molecule causing the hydration of rare earth ions, which weakens the complex bonds between CMPO and REEs. Consequently, water provides the highest stripping efficiency. In addition, water is an eco-friendly and low-cost reagent and was used for eluting REEs for the column proof-of-concept experiment.

### Effect of Desorption Time (Release Kinetics)

The release kinetics of sorbed REEs were investigated using ultrapure water and contact time from 30 to 120 min, as shown in Figure S5b. The desorption efficiency was reduced from 42.4 to 32.2% when the contact time was increased from 30 to 60 min, respectively. This phenomenon is likely an artifact of the batch experimental procedure, where eluted REEs are resorbed on the impregnated silica gel as the solution chemistry slowly evolves.

### Column Study

The breakthrough profiles for the loading and stripping phases of a packed-bed chromatography column are shown in Figure 8. In the loading phase (0–26 PV), all 16 rare earth elements (REEs) and thorium (Th) exhibited near-complete sorption (*C*/*C*_0_ ≈ 0), reflecting their retention in the column media. Breakthrough (defined as *C*/*C*_0_ > 0.5) occurred after 26 PV. Lutetium (Lu) was the first heavy element to break through (observed at 35 PV), followed sequentially by Yb (39 PV), Tm (40 PV), Y (43 PV), Er (44 PV), Ho (59 PV), Dy (74 PV), Tb (89 PV), Gd (96 PV), Eu (110 PV), La (114 PV), Sm (121 PV), Nd (128 PV), Pr (131 PV), and Ce (131 PV). Thorium (Th) and scandium (Sc) remained fully retained (*C*/*C*_0_ = 0) until the stripping phase began at ∼136 PV. The breakthrough sequence aligned with the selectivity of the media observed in batch experiments: Lu < Yb < Tm < Y < Er < Ho < Dy < Tb < Gd < Eu < La < Sm < Nd < Ce < Pr (Figure 8a).

**Figure 8:**
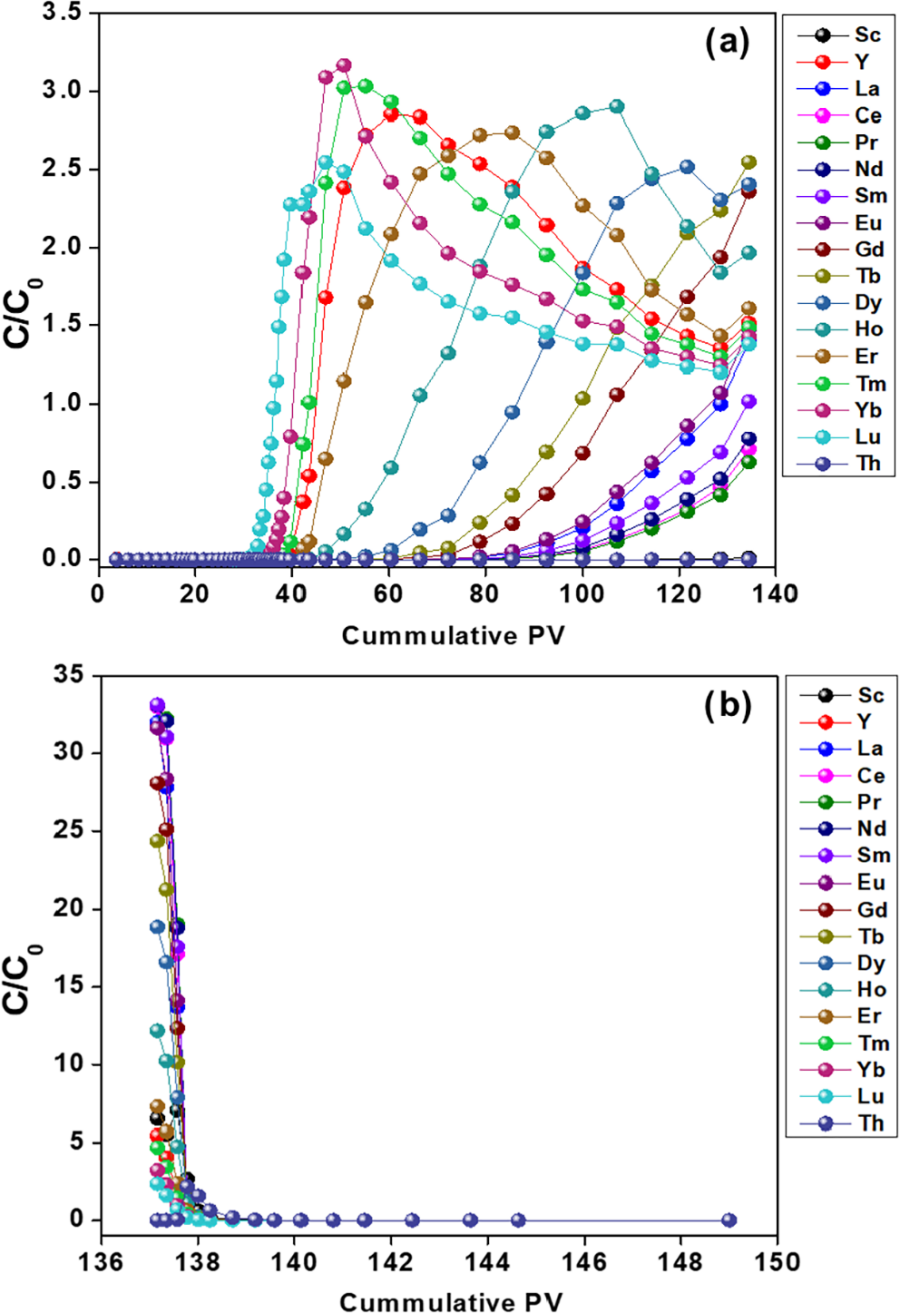
Breakthrough curves of the fixed-bed column experiment for (a) loading cycle and (b) strip cycle.

Chromatographic separation relies on competitive binding between sorbed REEs and incoming aqueous REEs. For the first 35 PV, the system has excess binding sites and all metal ions are sorbed. Once all sites are filled, the stronger media affinity for light REEs initiates substitution (desorption) of preadsorbed heavy REEs. The release of REEs can be observed when *C*/*C*_0_ > 1.0, as light REEs continue to sorb and heavy REEs previously sorbed are released. To evaluate desorption efficiency, the inlet solution was switched to ultrapure water after 136 PV. As shown in Figure 8b, over 95% of all elements were rapidly eluted within the first 15 PV of the stripping phase, highlighting efficient media regeneration.

The detailed fractionations of REEs during loading and stripping cycles are presented in Figures S6 and S7. Fractional collection and recombination can maximize the value of the selectivity and purity. For instance, fractions for PV 23–26 yielded 98% pure Lu, while PV 27–31 contained a Lu–Yb mixture (94% Lu, 5.5% Yb). Notably, 96% of collected loading-phase fractions were enriched in heavy REEs, underscoring the preference of the CMPO-impregnated silica gel media for retaining light REEs. This selectivity enables the effective separation and recovery of high-purity REEs under dynamic flow conditions.

A flow rate of 0.3 mL h^–1^ was used, which results in a residence time of about 20 min (∼3 bed volumes per hour). Sorption kinetics experiments also show that much higher flow rates could be used without a loss of performance. Based on the steep slopes found in the breakthrough curves shown in Figure 8 and the absence of a measurable breakthrough for all elements measured, increased flow rates for applications of the media can be anticipated.

### Proof-of-Concept with Rock Phosphate Leachate

An application study was conducted using a rock phosphate fertilizer (Falcon Isle Resources, UT), which has been shown to have >900 mg/kg REEs content in a previous study.^63,64^ The compositions of heavy elements and REEs are listed in Table S3. Although there were very high concentrations of major and heavy elements, the CMPO sorbent extracted 49% of La and 58% of Na, with minimal uptake of Y (8.4%) and negligible adsorption of heavy metals (Figure 9).

**Figure 9:**
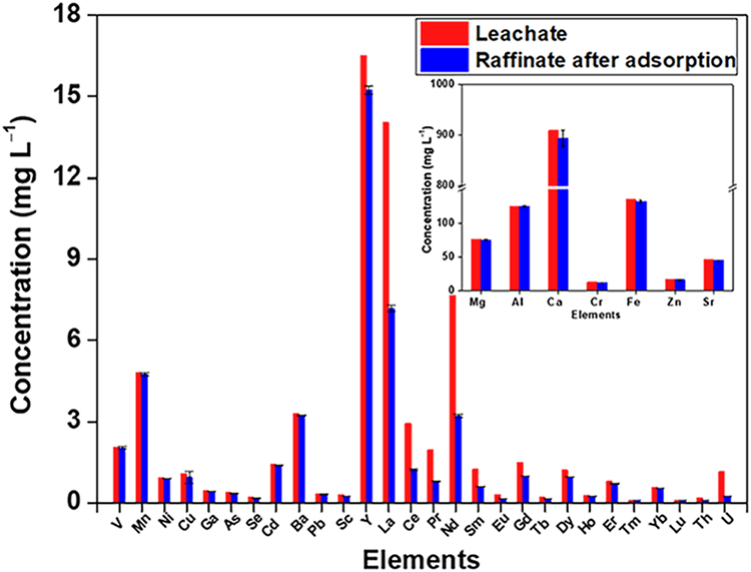
CMPO-impregnated silica gel sorption screening from a phosphate rock leachate (1.0 M HNO_3_, 4.0 h, 3 g L^–1^, 10 rpm, 25 °C).

The initial leachate has 3.6% (±0.003) rare earth elements based on polyvalent cation mass fraction analysis (Figure 10), while rare earth elements represent ∼64% of the polyvalent cation fraction in the sorbed fraction (over 20× enrichment in rare-earth elements). The neodymium fraction increased from 0.55% (±0.001) in the leachate to 16.5% (±0.80) in the sorbed fraction, equal to 30× enrichment.

**Figure 10:**
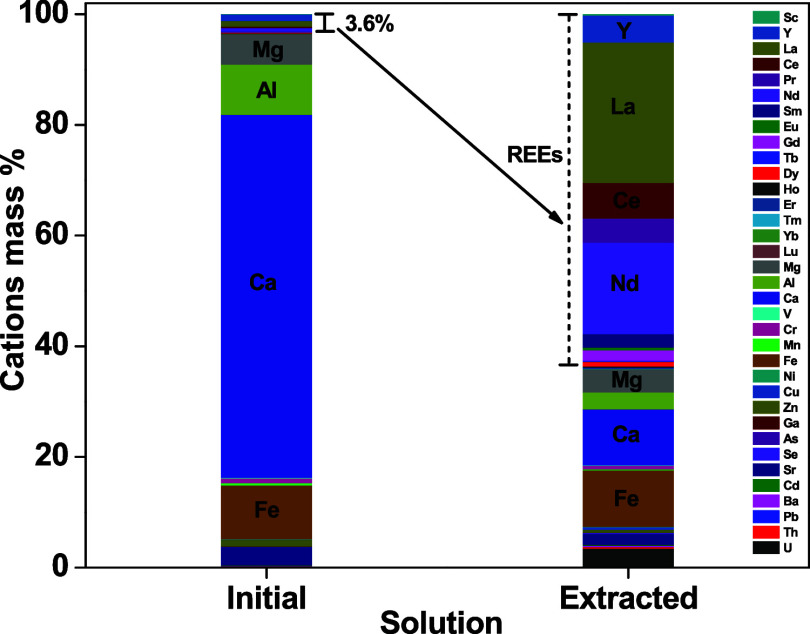
Polyvalent cation mass fraction (%) sorbed to CMPO-impregnated silica gel from a phosphate rock leachate based on analysis of 35 polyvalent cations (1.0 M HNO_3_, 4.0 h, 3 g L^–1^, 10 rpm, 25 °C).

### Environmental Implications

Rare-earth elements (REEs) are essential to several applications including high-performance magnets, fluorescent lighting, batteries, wind turbines, hybrid vehicles, and fluorescent lamps, among other advanced technologies. REEs exhibit similar chemical characteristics, posing challenges for their separation from liquid solutions.

A new ligand-associated CMPO-silica sorbent for rare-earth element separations was synthesized, characterized, and tested in a proof-of-concept column system by immobilizing octyl-phenyl-*N*,*N*-diisobutyl carbamoyl methyl phosphine oxide (CMPO) on a silica solid support. Batch adsorption experiments reveal that the CMPO-impregnated silica gel has high selectivity toward light REEs, with marginal uptake of heavy REEs, and almost negligible sorption of heavy metals in a mixed 46-element solution. Kinetic studies show equilibrium is reached in <4 h and follow pseudo-second-order. The Langmuir isotherm adsorption behavior supports a monolayer chemisorption mechanism with an uptake capacity of 0.0042 mmol of REEs per 0.0178 mmol of CMPO in the sorbent, suggesting the formation of a 1:4 metal–ligand complex of REE­(NO_3_)_3_.4­(CMPO). Ultrapure water attained the highest elution efficiency of sorbed REEs on the CMPO-impregnated silica gel.

A proof-of-concept column separation highlights the practical applicability of CMPO-impregnated silica gel sorbent to be used as a “hold-back” for light REEs, enabling efficient separation of the more valuable heavy REEs. The breakthrough curve indicates that the collected loading-phase fractions contained highly enriched heavy REEs (96.28%), while the CMPO-impregnated silica gel media retained the light REEs. The media was used to increase the divalent cation mass fraction from <3% rare earth elements to ∼64% in the sorbed fraction in a complex phosphate rock leachate. Further EXAFS experiments in combination with vibrational and luminescence studies would be of strategic importance to further understand the coordination chemistry of CMPO and REEs.

## Supplementary Material



## Notes

Experimental details, methods, and results, including scanning electron microscopy (SEM) images and elemental abundance for individual REEs in loading and stripping solutions from the column experiment (PDF)

## #

Department of Civil Engineering, Indian Institute of Technology Hyderabad, Telangana, 502284, India
