# Supplementary material for: CMPO-Functionalized Silica Sorbents for pH-Tunable Separation and Enrichment of Rare-Earth Elements from Environmental Matrices
Source: ACS Sustain Chem Eng. 2026 Feb 16;14(8):3946–57. doi: 10.1021/acssuschemeng.5c11109 (PMC12958340; doi:10.1021/acssuschemeng.5c11109)
Supplement: Supplementary file 1 [file sc5c11109_si_001.pdf]

Supporting Information

for

CMPO-Functionalized Silica Sorbents for pH-Tunable Separation and Enrichment of Rare Earth Elements from Environmental Matrices

Ahmed K. Sakr,[1,]* Sai Praneeth,[1] Preetom Kishore Roy, Timothy M. Dittrich*

Department of Civil and Environmental Engineering, Wayne State University, 5050 Anthony Wayne Drive, Detroit, MI 48202, USA

*Corresponding authors – ahmed.sakr@wayne.edu (A. K. Sakr)
timothy.dittrich@wayne.edu (T. M. Dittrich)

[1]Co-first authors (equal contribution)

The file includes

Number of pages: 14

Number of figures: 7

Number of tables: 3

Text

Text S1. CMPO ligand baseline characterization - solvent extraction (SX).................................4

Text S2. Batch Sorption experiments (capacity, kinetics, solid support type, 1-5 M acid range, 43 element competition) 4

Text S3. Desorption experiments 5

Figures

Figure S1. Sorption of REEs on silica gel (1.0 M HNO3, 102 mg L^{-1} REEs+Th, 24 h, 3 g L^{-1}, 10 rpm, 25 °C)………………………………………………………………...……...…………6

Figure S2. a) SEM imaging of the silica gel particle at × 250 and b, c) EDS image and spectrum of two random sampling data points at the same × 250 with elemental compositions……………7

Figure S3. a) SEM imaging of the CMPO- silica gel media at × 250 and b-d) EDS image and spectrum of three random sampling data points at the same × 250 media with elemental compositions………………………………………………………………………………8

Figure S4. Effect of acid on the extraction of REEs (1.0 M, 102 mg L−1 REEs+Th, 15 min, 1:1 A/O ratio, 50 rpm, 25 °C, CMPO/kerosene)…………………………………………………9

Figure S5. Desorption process REEs sorbed on the CMPO-impregnated silica gel media using a) different eluents (1.0 M eluent, 1:40 S/L ratio, 30 min, 10 rpm, 25 °C) and b) different desorption time (H_2O, 1:40 S/L ratio, 10 rpm, 25 °C)...10

Figure S6. Purity of REEs (% of total) for fixed-bed column experiment in terms of individual element in column effluent groups, where loading cycle samples are represented in seven groups of combined PV samples and the stripping cycle samples are combined into five PV groups.....11

Figure S7. Purity of REEs (% of total) for fixed-bed column experiment in terms of heavy and light REEs groups in loading and stripping effluent. 12

Tables

Table S1. Kinetic models of REEs sorbed on the CMPO-impregnated silica gel. 13

Table S2. Isotherm models of REEs sorbed on the CMPO-impregnated silica gel.....................13

Table S3. Chemical composition of leachable material from phosphate fertilizer based on ICP-MS analysis……………………………………………………………...……………………14

Text S1. CMPO ligand baseline characterization - solvent extraction (SX)

The following equations were used to calculate the distribution ratio (D) and extraction efficiency (E, %):

$$D = \frac{C_o}{C_a} \quad (1)$$

$$E\,(\%) = \frac{\mathrm{D}}{\mathrm{D}+(\frac{V_a}{V_o})} \times 100 \quad (2)$$

where C_o and C_a (mg L^{-1}) represent the equilibrium REE ion concentrations in the organic and aqueous phases, respectively. V_o and V_a (mL) refer to the volume of organic and aqueous solutions, respectively.

Text S2. Batch Sorption experiments (capacity, kinetics, solid support type, 1-5 M acid range, 43 element competition)

The pseudo-first model is represented by using the equation: $q_t = q_e(1 - exp^{-k_1 t})$ (5)

where q_e (mg g^{-1}) is the adsorption capacity at equilibrium, q_t (mg g^{-1}) is the adsorption capacity at time t, t (min) is the contact time, and k_1 (min^{-1}) is the rate constant of pseudo-first-order adsorption.

The pseudo-second-order model is represented by using the equation: $q_t = \frac{k_2 {q_e}^2 t}{1+k_2 q_e t}$

(6)

where q_e (mg g^{-1}) is the adsorption capacity at equilibrium, q_t (mg g^{-1}) is the adsorption capacity at time t, k_2 (g mg^{-1} min^{-1}) is the rate constant of pseudo-second order adsorption, and t (min): contact time.

Adsorption isotherm experiments were conducted at 3.0 g L^{-1} of dosage and 6 hours of equilibrium time with a range of individual REE concentrations (1, 3, 6, 9, 12, 15, and 20 mg L^{-1}) and modeled for Langmuir and Freundlich adsorption models described below:

Langmuir sorption modeling: $q_e = \frac{q_m K_L C_e}{1+K_L C_e}$ (7)

Where q_e and q_m (mg g^{-1}) refer to the amount of metal ion adsorbed at equilibrium and complete monolayer adsorption capacity, respectively. C_e (mg L^{-1}) denotes the equilibrium concentration and K_L (L mg^{-1}) is the Langmuir adsorption constant.

Freundlich sorption modeling: $q_e = K_F C_e^{1/n}$ (8)

Where n represents the empirical parameter relating to the adsorption intensity, which varies with the heterogeneity of the material (dimensionless) and K_F ($L^{1/n}$ $mg^{(1-1/n)}$ g^{-1}) stands for the Freundlich adsorption constant.

Text S3. Desorption experiments

A desorption study was performed to extract the REEs sorbed on the CMPO-impregnated silica gel. Different eluents of H_2O, 1.0 M NaOH, 1.0 M NH_4OH, and 1.0 M $NaHCO_3$ were tested. The experiments were conducted using 1:40 solid-to-liquid (S/L) ratio of 300 mg of REEs-CMPO-impregnated silica gel and 12 mL of eluent for 30 min at 25 °C and 10 rpm. The effect of elution time was investigated by varying the time from 30 to 120 min. Each experiment was duplicated; the average and standard deviation were calculated and presented.

The desorption efficiency (*D*es, %) for all experiments was determined using the below equation:

$Des\ (\%) = \frac{C_d V_d}{m q_e} \times 100$ (9)

where C_d (mg L^{-1}) refers to the concentration of desorbed REEs, V_d (L) signifies the volume of eluent, m (g) represents the mass of material, q_e (mg g^{-1}) denotes the amount of metal ions adsorbed at equilibrium.

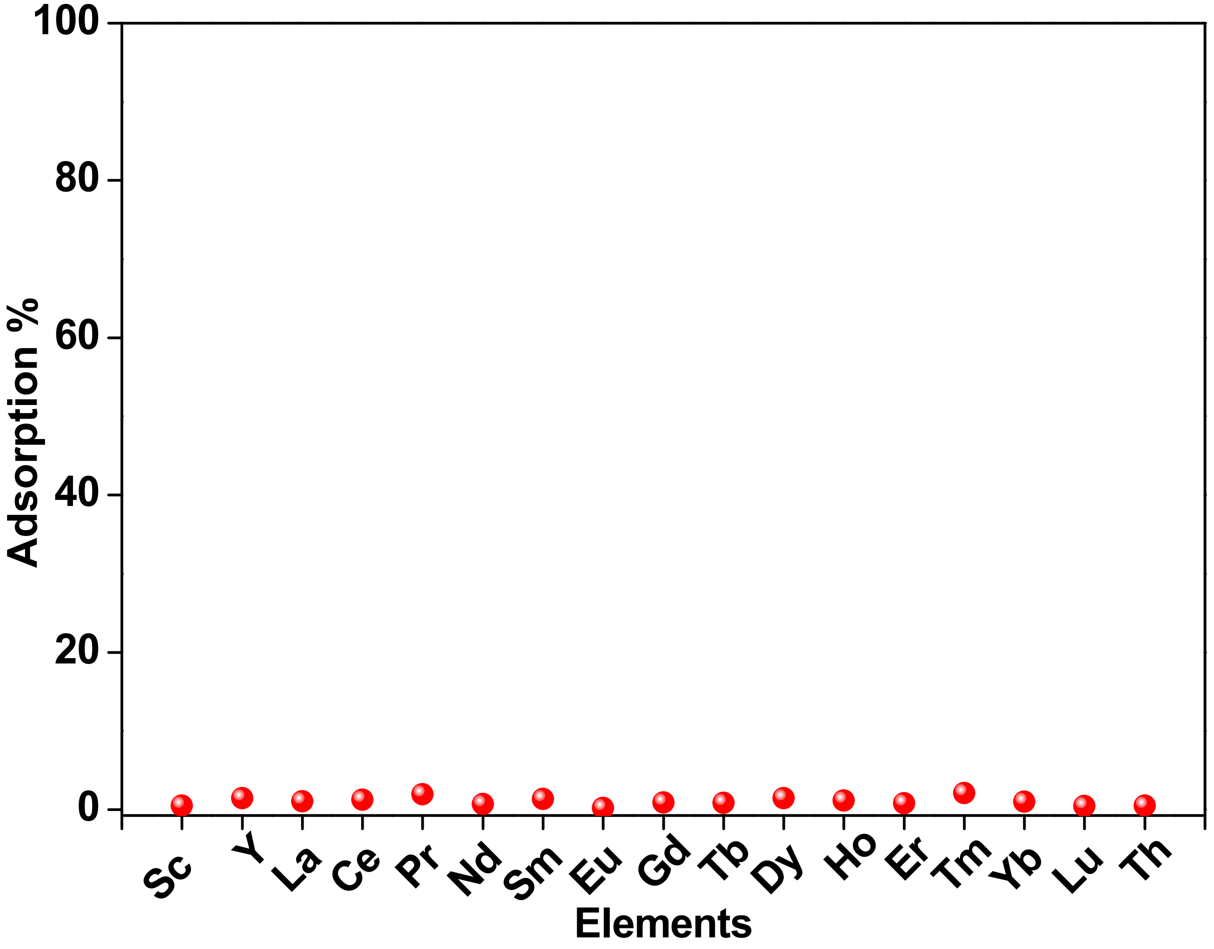

Figure S1. Sorption of REEs on silica gel (1.0 M HNO_3, 102 mg L^{-1} REEs+Th, 24 h, 3 g L^{-1}, 10 rpm, 25 °C).

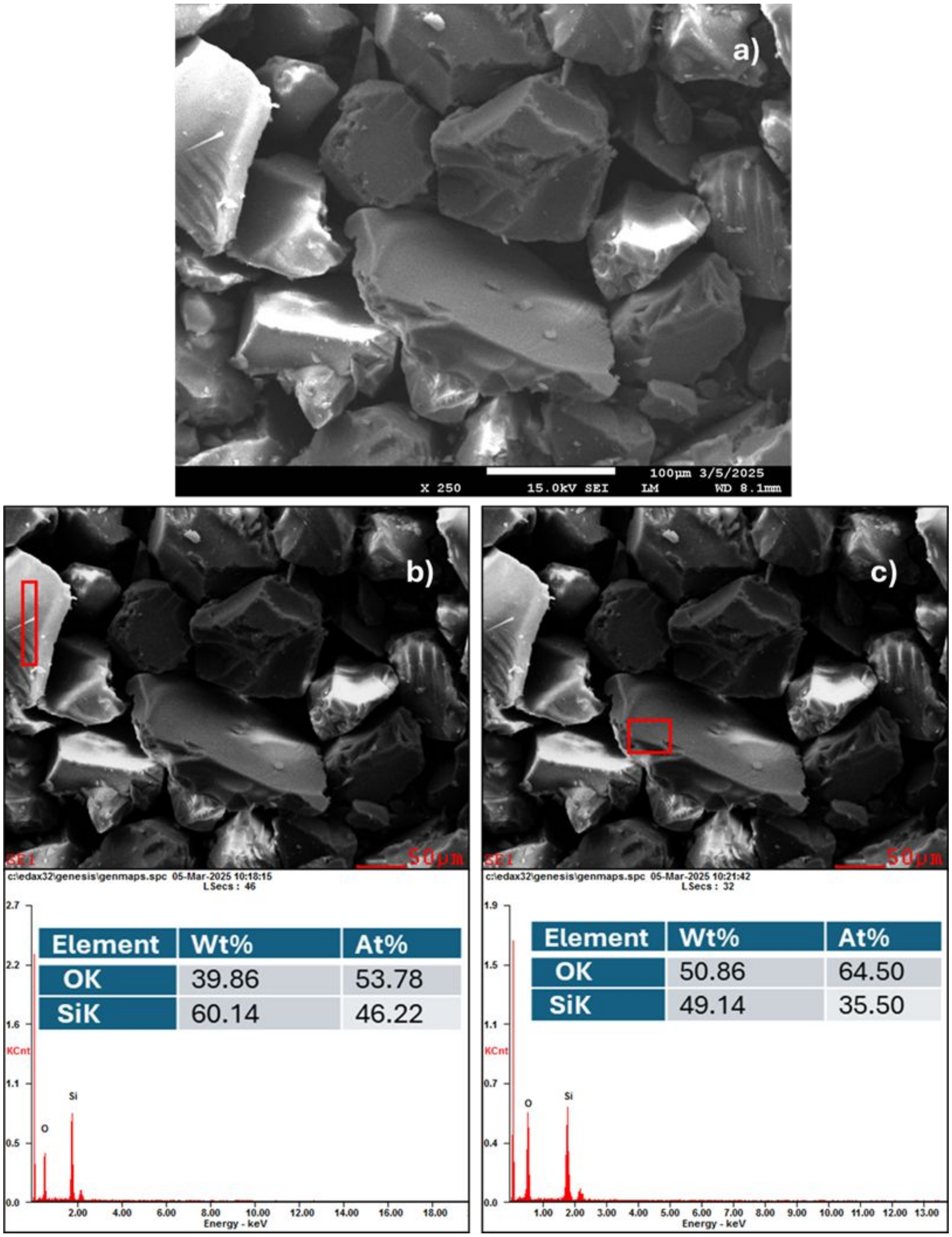

Figure S2. a) SEM imaging of the silica gel particle at × 250 and b, c) EDS image and spectrum of two random sampling data points at the same × 250 with elemental compositions.

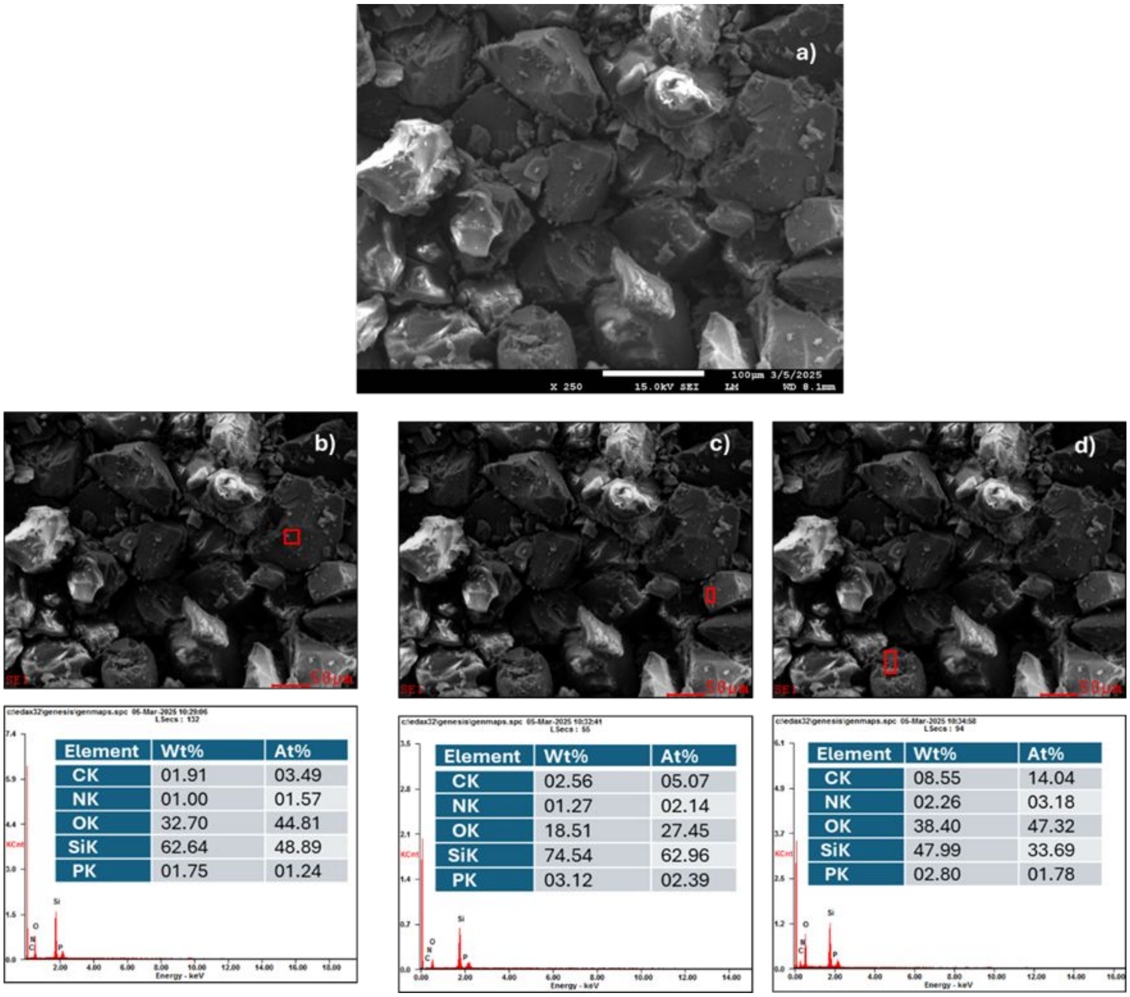

Figure S3. a) SEM imaging of the CMPO- silica gel media at × 250 and b-d) EDS image and spectrum of three random sampling data points at the same × 250 media with elemental compositions.

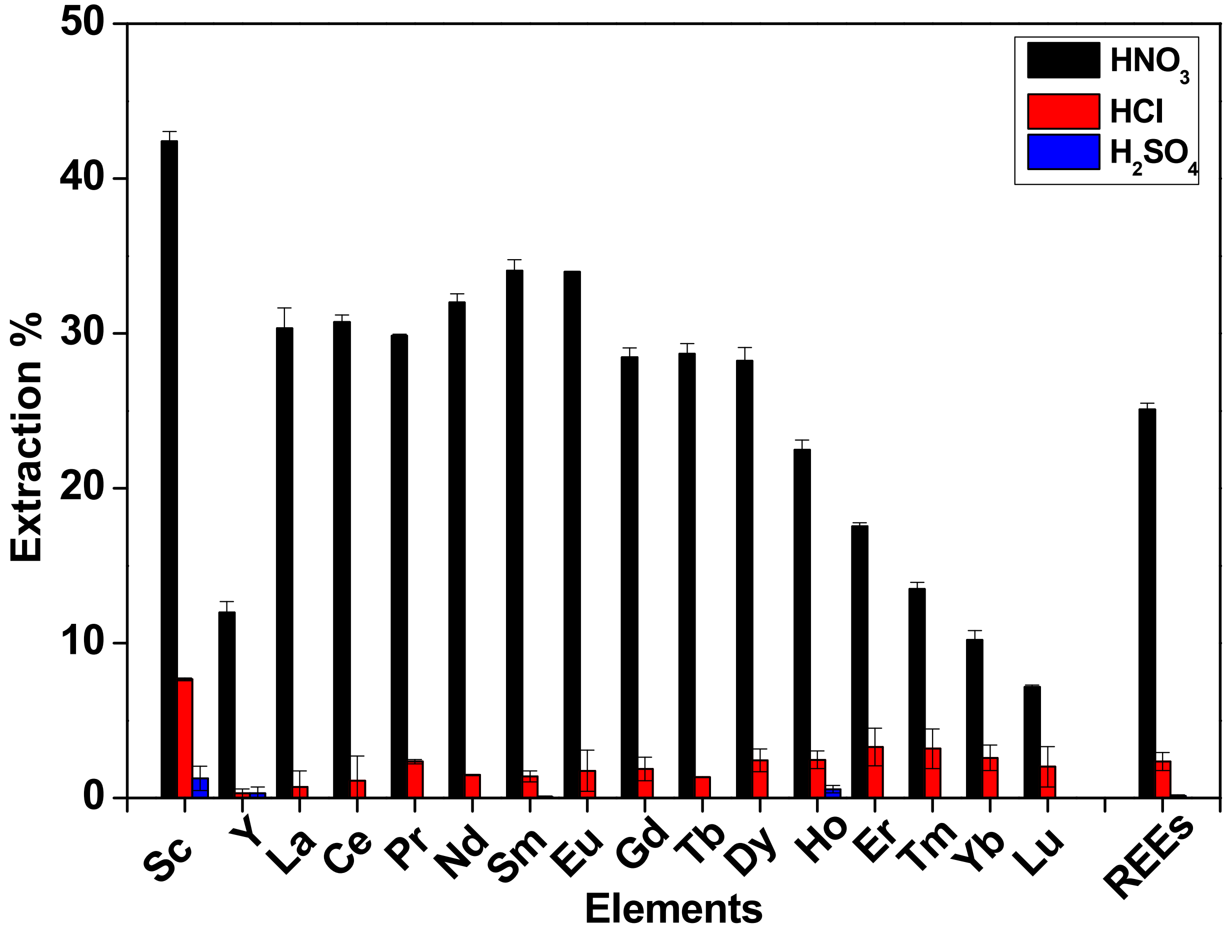

Figure S4. Effect of acid on the extraction of REEs (1.0 M, 102 mg L^{-1} REEs+Th, 15 min, 1:1 A/O ratio, 50 rpm, 25 °C, CMPO/kerosene).

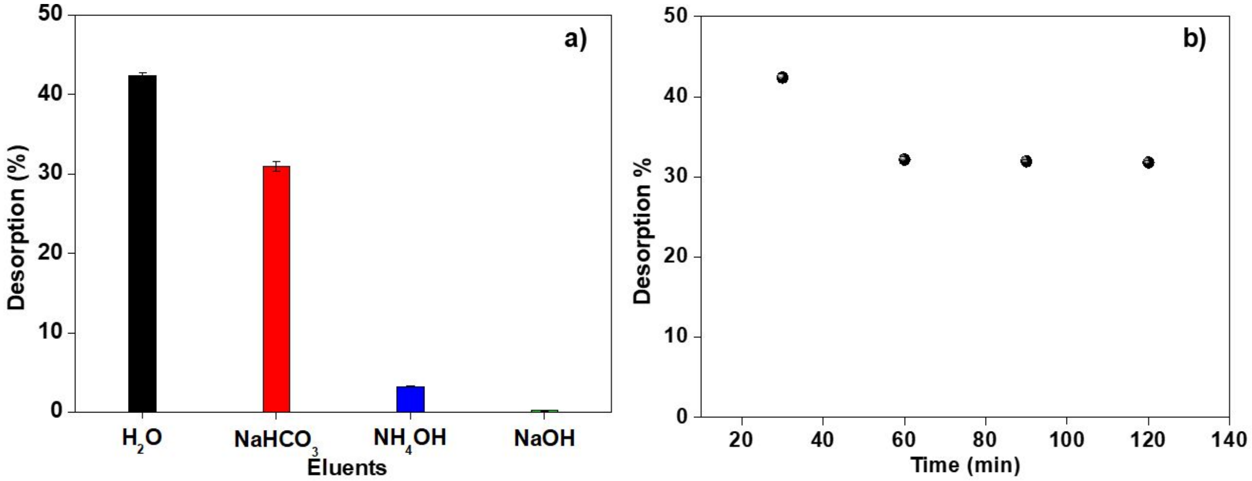

Figure S1. Desorption process REEs sorbed on the CMPO-impregnated silica gel media using a) different eluents (1.0 M eluent, 1:40 S/L ratio, 30 min, 10 rpm, 25 °C) and b) different desorption time (H_2O, 1:40 S/L ratio, 10 rpm, 25 °C).

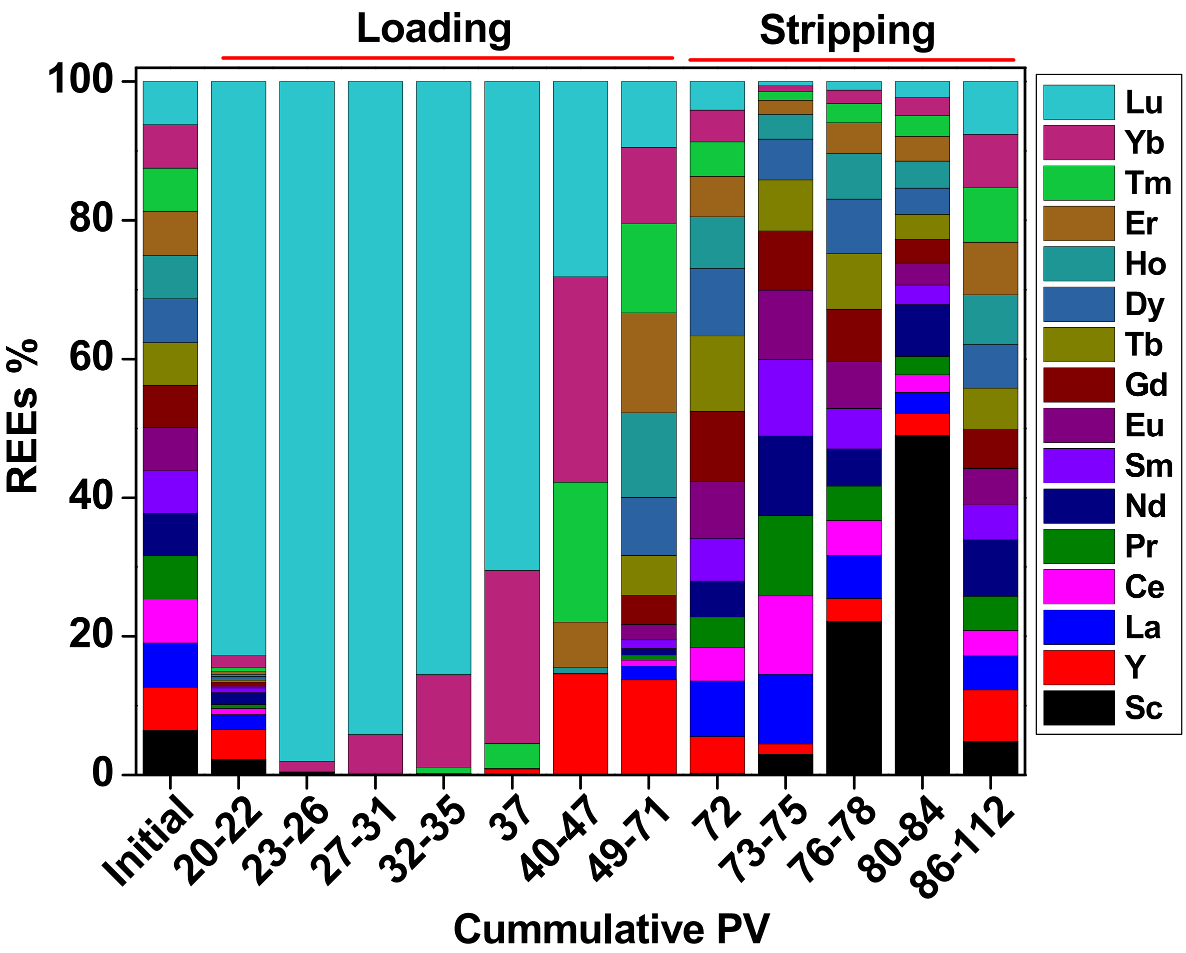

Figure S2. Purity of REEs (% of total) for fixed-bed column experiment in terms of individual element in column effluent groups, where loading cycle samples are represented in seven groups of combined PV samples and the stripping cycle samples are combined into five PV groups.

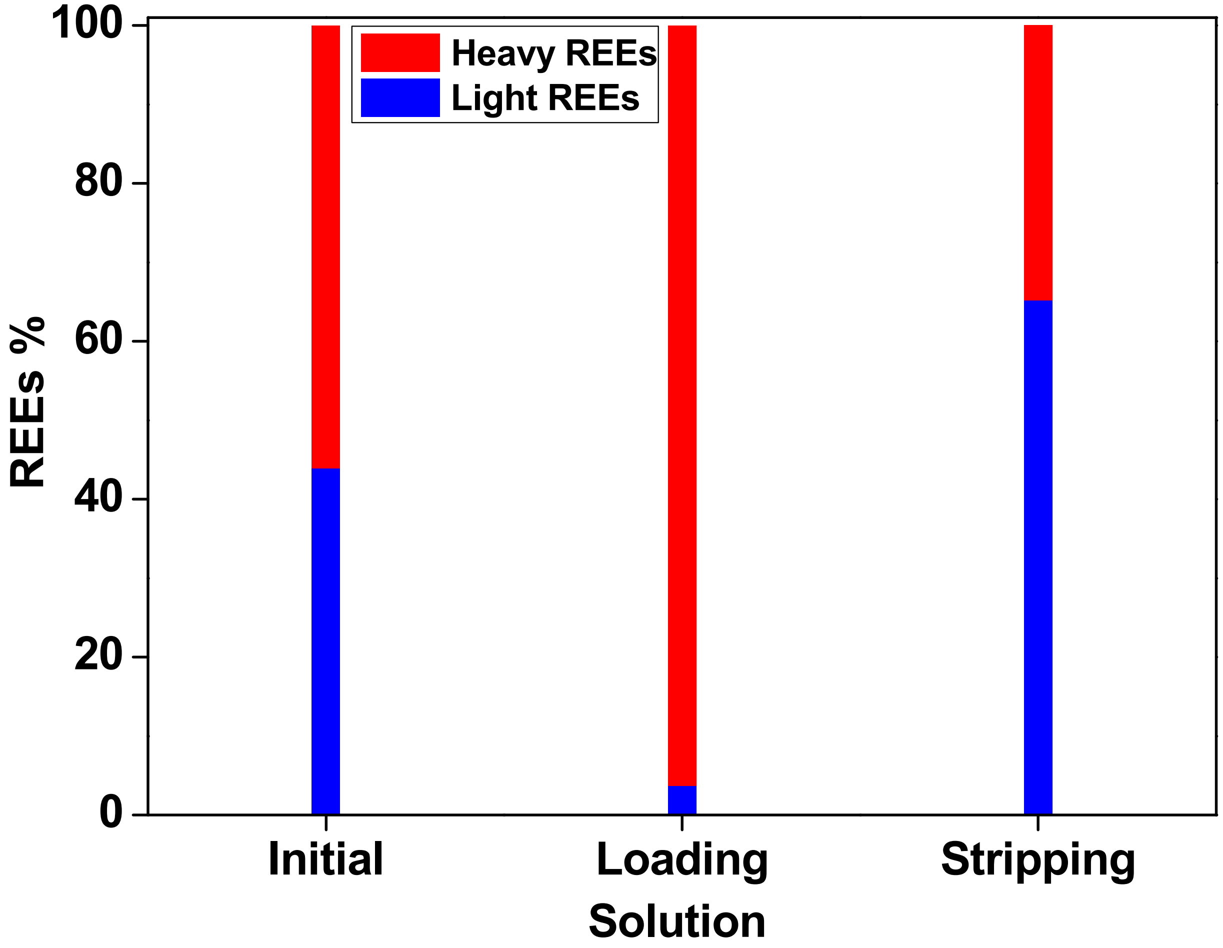

Figure S3. Purity of REEs (% of total) for fixed-bed column experiment in terms of heavy and light REEs groups in loading and stripping effluent.

Table S1. Kinetic models of REEs sorbed on the CMPO-impregnated silica gel.

	X^2	R^2	q_e (mg g^{-1})	k_1 (min^{-1})
**PFO**	387.92	0.81	8.23	0.32
	**X^2**	**R^2**	**q_e (mg g^{-1})**	**k_2 (g mg^{-1} min^{-1})**
**PSO**	96.64	0.95	8.43	0.05

Table S2. Isotherm models of REEs sorbed on the CMPO-impregnated silica gel.

	X^2	R^2	q_m (mg g^{-1})	K_L	R_L
**Langmuir**	2389.02	0.99	13.35	0.05	0.059
	**X^2**	**R^2**	**K_F (L$^{1/n}$ mg$^{1-(1/n)}$ g^{-1})**	**n**	
**Freundlich**	28817.67	0.88	1.29	1.89	

Table S3. Chemical composition of leachable material from phosphate fertilizer based on ICP-MS analysis.

Elements	Concentration (mg kg^{-1})	REE	Concentration (mg kg^{-1})
Mg	1528.58 ± 68.9	Sc	5.85 ± 0.58
Al	2508.96 ± 113.24	Y	330.18 ± 14.9
Ca	18175.06 ± 120.32	La	280.73 ± 12.67
Cr	246.55 ± 11.13	Ce	58.78 ± 2.66
Fe	2703.32 ± 122.01	Pr	39.13 ± 1.77
Zn	322.68 ± 14.56	Nd	153.37 ± 6.92
Sr	914.12 ± 14.26	Sm	24.71 ± 1.12
V	41.12 ± 1.86	Eu	5.85 ± 0.26
Mn	96.32 ± 4.35	Gd	29.96 ± 1.35
Ni	18.40 ± 0.83	Tb	3.96 ± 0.18
Cu	21.33 ± 3.09	Dy	24.20 ± 1.09
Ga	8.72 ± 0.39	Ho	5.57 ± 0.25
As	7.50 ± 0.34	Er	15.85 ± 0.72
Se	4.0 ± 0.0	Tm	2.01 ± 0.09
Cd	28.46 ± 1.28	Yb	11.50 ± 0.52
Ba	65.96 ± 3.0	Lu	1.80 ± 0.08
Pb	6.60 ± 0.30	REEs	993.44 ± 43.99
Th	3.6 ± 0.16		
U	23.21 ± 1.05		
